# Use of hemoglobin A1c to identify dysglycemia in cystic fibrosis

**DOI:** 10.1371/journal.pone.0250036

**Published:** 2021-04-21

**Authors:** Amy Darukhanavala, Filia Van Dessel, Jannifer Ho, Megan Hansen, Ted Kremer, David Alfego

**Affiliations:** 1 Department of Pediatric Endocrinology, University of Massachusetts Medical Center, Worcester, MA, United States of America; 2 University of Massachusetts Medical School, Worcester, MA, United States of America; 3 Department of Pediatric Pulmonology, University of Massachusetts Medical Center, Worcester, MA, United States of America; Università degli Studi di Milano, ITALY

## Abstract

**Background:**

Cystic fibrosis (CF) leads to pancreatic endocrine dysfunction with progressive glycemic disturbance. Approximately 30%–50% of people with CF eventually develop CF–related diabetes (CFRD). Pre-CFRD states progress from indeterminant glycemia (INDET) to impaired fasting glucose (IFG) or impaired glucose tolerance (IGT). Screening guidelines recommend inconvenient annual 2-hour oral glucose tolerance tests (OGTTs), beginning at age 10 years. More efficient methods, such as hemoglobin A1C (HbA1c), have been evaluated, but only limited, relatively small studies have evaluated the association between HbA1c and pre-CFRD dysglycemic states.

**Objective:**

To determine whether HbA1c is an appropriate screening tool for identifying patients with pre-CFRD dysglycemia to minimize the burden of annual OGTTs.

**Methods:**

This retrospective review evaluated medical records data of all University of Massachusetts Memorial Health System CF patients with an HbA1c result within 90 days of an OGTT between 1997 and 2019. Exclusion criteria were uncertain CF diagnosis, other forms of diabetes, or incomplete OGTT. In total, 56 patients were included and categorized according to OGTT results (American Diabetes Association criteria): normal glucose tolerance, INDET, IFG, or IGT. Associations were evaluated between HbA1c and OGTT results and between HbA1c and pre-CFRD dysglycemic states.

**Results:**

Mean HbA1c was not significantly different between patients with normal glucose tolerance and those in the INDET (p = 0.987), IFG (p = 0.690), and IGT (p = 0.874) groups. Analysis of variance confirmed the lack of association between HbA1c and glycemia, as mean HbA1c was not significantly different amongst the four categories (p = 0.250).

**Conclusion:**

There is increasing awareness of the impact of pre-CFRD states, including reduced pulmonary function and nutritional status. Unfortunately, our results do not support using HbA1c as a screening tool for pre-CFRD dysglycemia, specifically INDET, IFG, and IGT. Further studies are warranted to evaluate more efficient screening methods to reduce the burden of annual OGTTs.

## Introduction

Cystic fibrosis (CF) affects 1 in 2500–3000 live births and is the most common life-threatening genetic disease in Caucasians. CF is caused by an autosomal recessive mutation in the CF transmembrane conductance regulator (*CFTR*) gene, which leads to viscous secretions in multiple organs, especially the lungs [1]. Viscous secretions in the pancreas produce exocrine dysfunction, presenting as pancreatic insufficiency in up to 90% of people with CF. Inflammation and destruction of insulin-producing islet beta cells cause pancreatic endocrine dysfunction, resulting in progressive glycemic disturbance [2]. Approximately 40%–50% of people with CF will eventually develop CF-related diabetes (CFRD), with 20% diagnosed in adolescence [3].

The primary cause of CFRD is insulin deficiency. Although not autoimmune in nature, CFRD is similar to type I diabetes, which is also due to insulin deficiency. However, various clinical conditions may promote insulin resistance in CFRD, similar to type II diabetes [4, 5]. The presence of CFRD has been demonstrated to worsen clinical outcomes in patients with CF through declining pulmonary function and nutritional status, resulting in increased mortality [6–11].

In CF, the glycemic disturbance often progresses over decades before eventually culminating in CFRD. Initially, blunting of the first-phase insulin response results in postprandial hyperglycemia. This is followed by continued decline in insulin secretion, leading to the eventual diagnoses of indeterminant glycemia (INDET), impaired fasting glucose (IFG) or impaired glucose tolerance (IGT) [1, 8, 12, 13]. While the effects of CFRD on clinical outcomes are well understood, emerging evidence suggests that these preceding intermediate dysglycemic states have a substantial negative impact on lung function, weight, and even mortality [7, 13–15].

Early detection and treatment of dysglycemia and CFRD are crucial for reducing deterioration of pulmonary function and nutritional status. Current screening guidelines for CFRD include an annual 2-hour oral glucose tolerance test (OGTT) beginning at age 10 years. However, the CF Foundation Patient Registry 2019 Annual Data Report noted that only 51.75% of individuals with CF are compliant with the recommendation to undergo this inconvenient and time-consuming test. As a result, more convenient and efficient methods to identify CFRD, such as hemoglobin A1C (HbA1c), have been evaluated. Studies thus far have shown poor correlation between HbA1c and CFRD [2, 9, 10, 16, 17]. However, no published studies have evaluated the association between HbA1c and pre-CFRD dysglycemic states, such as INDET, IFG, and IGT.

The hypothesis of this study is that annual OGTTs may be an unnecessary screening tool for detecting glycemic dysregulation in patients with CF. Our objective was to determine whether HbA1c, an effective and efficient measure of glycemic control, is a suitable screening tool to identify patients with possible pre-CFRD dysglycemic states, which could be subsequently evaluated with a 2-hour OGTT. If deemed a successful screening method, this would minimize the substantial burden associated with an annual OGTT, potentially increasing early detection of these pre-CFRD states.

## Methods

This study was conducted at the University of Massachusetts (UMass) Memorial Medical Center in Worcester, Massachusetts. Established in 1985, the UMass Memorial Cystic Fibrosis Center was accredited by the national Cystic Fibrosis Foundation in 1993 and offers a team approach to adult and pediatric care, including health care providers, registered dieticians, respiratory therapists, pharmacists, an endocrinologist, a social worker, and a psychologist. The center currently serves 125 patients with CF in central Massachusetts. The protocol was approved by the Institutional Review Board of the University of Massachusetts Medical School (no: H00016591).

### Study design

This study was a retrospective review of UMass Memorial Health System electronic medical records between 1997 and 2019. This involved reviewing Epic, our current electronic medical record system, as well as previous systems, such as Pulsecheck. We used ICD-9 or ICD-10 codes for CF to identify all pediatric and adult patients treated at UMass during the study time period and further narrowed the search to include only CF patients with an HbA1c result within 90 days of an OGTT. All data was fully anonymized before it was accessed and the IRB waived the requirement for informed consent.

### Subjects

The inclusion criteria were as follows: any age group; documented diagnosis of CF; and an HbA1c and a complete 2-hour OGTT obtained no more than 90 days apart. Patients were excluded from the study if the diagnosis of CF was uncertain; they had other forms of diabetes (e.g., type 1 or type 2 diabetes); they had cognitive impairment; they were pregnant or incarcerated; or the OGTT was incomplete (i.e., it did not include glucose values at all three timepoints: 0, 1, and 2 hours). In total, 56 patients met our inclusion and exclusion criteria.

### Assessments

HbA1c was measured during routine clinic visits by obtaining a standard blood sample, followed by separation and quantification of glycated and non-glycated hemoglobin. The test is an indicator of the average blood glucose over the preceding 90 days. The 2-hour OGTT was performed after a minimum fast of 8 hours. First, a baseline fasting plasma glucose was obtained (at 0 hours), followed by ingestion of a drink containing 75 g of glucose (in children, the drink contained 1.75 g of glucose/kg to a maximum of 75 g). Additional plasma glucose measurements were obtained 1 hour and 2 hours after consuming the drink. The patients were categorized into four groups based on OGTT results, in accordance with American Diabetes Association criteria: normal glucose tolerance, INDET, IFG, and IGT (Table 1).

**Table 1:** Classification of glycemic states based on oral glucose tolerance test results.

Classification	Fasting plasma glucose (mg/dL)	AND/OR criteria	One-hour plasma glucose (mg/dL)	Two-hour plasma glucose (mg/dL)
Normal glucose tolerance	< 100	AND	--	< 140
Indeterminate glycemia (INDET)	< 100	AND	≥ 200	< 140
Impaired fasting glucose (IFG)	100–125	AND	--	< 140
Impaired glucose tolerance (IGT)	< 126	AND	--	140–199
Diabetes	≥ 126	AND/OR	--	≥ 200

### Statistical analysis

First, the association between HbA1c and OGTT results was evaluated. Pearson correlation analysis was used to assess the relationship between HbA1c and each of the three OGTT glucose values (0, 1, and 2 hours). We also performed univariate linear regression to identify individual associations between HbA1c and glucose levels at each time during the OGTT test. Times with statistically significant results in univariate analysis were evaluated by multivariate regression analysis to assess combined effects.

Next, the association between HbA1c and the glycemic states was assessed. Student’s t-test was used to compare mean HbA1c in the normal glucose tolerance group with mean HbA1c in each of the three dysglycemia groups. Analysis of variance (ANOVA) was used to compare mean HbA1c amongst all four groups. Microsoft Excel and SPSS for Windows were used for all statistical analyses. P values <0.05 were considered statistically significant.

## Results

The study included 56 subjects: 22 females and 34 males. The mean age of subjects was 15.4 years (SD ±12.7) (range 7–63 years). Overall, 90.6% of patients were Caucasian, and 81.2% were diagnosed with pancreatic insufficiency. Normal glucose tolerance was present in 34 patients. The remaining patients were diagnosed with dysglycemia: INDET, 6 patients; IFG, 7 patients; and IGT, 9 patients.

In Pearson correlation analysis, HbA1c was positively correlated with blood glucose values at 0 hour (r = 0.248), 1 hour (r = 0.219), and 2 hours (r = 0.369) of the OGTT, although the low r values indicate that these associations were weak. Linear regression models of individual time points revealed statistically significant associations between HbA1c and fasting glucose (p = 0.037) and 2-hour glucose (p = 0.0008), although the relationships were weak (both R^2^ <0.2). HbA1c was not significantly associated with 1-hour glucose (p = 0.104). In multivariate regression analysis of fasting glucose and 2-hour glucose, only the 2-hour glucose remained significantly associated with HbA1c (p<0.001), although this relationship was also weak.

Mean HbA1c was not significantly different between patients with normal glucose tolerance and those in the INDET (p = 0.987), IFG (p = 0.690), and IGT (p = 0.874) groups (Table 2). ANOVA confirmed the lack of association between HbA1c and glycemia, as there was no significant difference in mean HbA1c amongst the four categories (p = 0.250).

**Table 2:** Hemoglobin A1c values of the four glycemic state groups.

	NGT	INDET	IFG	IGT
**HbA1c, mean**	5.6	5.6	5.5	5.5
**HbA1c, SD**	0.46	0.50	0.41	0.45
**n**	34	6	7	9
**P-value**	–	0.987	0.690	0.874

P-values are compared to the normal glucose tolerance group (Student’s t-test).

HbA1c, hemoglobin A1c; INDET, indeterminate glycemia; IFG, impaired fasting glucose; IGT, impaired glucose tolerance; NGT, normal glucose tolerance; SD, standard deviation.

## Discussion

Our results do not support the use of HbA1c as a possible screening tool for pre-CFRD dysglycemic states, specifically INDET, IFG, and IGT. There is increasing awareness of the impact of these pre-diabetic states on the CF population, including reduced pulmonary function and nutritional status [7, 13–15]. Before the development of frank diabetes, insulin secretion is delayed and blunted, leading to prolonged states of hyperglycemia that may contribute to increased bacterial growth, inflammation and catabolic wasting [1, 7, 15]. Hameed et al. reported that CFRD is a late event and that preceding glucose abnormalities were associated with a decline in lung function and body mass index (BMI) during the previous year [6]. A retrospective study by Lanng et al, evaluating 38 children and adults with CFRD, reported a gradual decline in BMI and pulmonary function up to 4 years before the diagnosis of CFRD [7]. Additionally, Brodsky et al. reported that the INDET dysglycemic state was associated with worsening pulmonary function [18].

Although it is well known that CFRD increases morbidity and mortality in the CF population, emphasizing the need for early detection and treatment, there is a paucity of data regarding screening for preceding dysglycemia in these patients. Current screening methods for CFRD and dysglycemia require undergoing the cumbersome, time consuming, and burdensome 2-hour OGTT on an annual basis. As patients with CF are already subjected to frequent clinic visits, investigations, and hospitalizations, it is understandable that they are often frustrated with the prospect of another prolonged fasting test to potentially diagnose an additional disease state. The OGTT screening rate at our institution, although higher than the national average reported by the CF Foundation, remains dishearteningly low, suggesting that there may be many patients with undiagnosed pre-CFRD or CFRD. Additional methods to identify CFRD, such as continuous glucose monitoring (CGM), have proven equally difficult to implement because of the burden of obtaining a CGM device and the need to constantly wear the device.

HbA1c is a simple test for assessing glycemic control, and it can even be used to diagnose type I or type II diabetes. Although previous research indicated that HbA1c has low sensitivity and specificity for diagnosing CFRD [2, 9, 10, 16, 17], no study has evaluated the association between HbA1c and pre-CFRD dysglycemic states. Because of early loss of the first phase insulin response, the OGTT of CF patients without CFRD often reveals major abnormalities in early insulin secretion [1, 18, 19]. Our study aimed to determine whether HbA1c was able to identify INDET, IFG, and IGT states and whether there was a relationship between HbA1c and any of the three OGTT glucose values that could help detect these dysglycemic states. Although we found a small positive correlation between HbA1c and glucose at each OGTT stage and a statistically significant, but weak, association between HbA1c and 2-hour OGTT glucose during multivariate analysis, our results overall do not support the use of HbA1c as a screening tool for pre-CFRD dysglycemic states.

The poor association between HbA1c and CFRD or dysglycemic states may be attributed to HbA1c being spuriously low because of increased red blood cell turnover secondary to inflammation. Additionally, the transient postprandial hyperglycemia may not significantly affect the glycosylation of red blood cells [20]. Furthermore, other possible CF co-morbidities, such as iron deficiency anemia and severe chronic liver disease, may interfere with HbA1c measurements [2]. Some studies have suggested that lower cutoff values may improve the sensitivity and specificity of the test [2], but data are limited, and the 2009 updated Clinical Care Guidelines from the American Diabetes Association and Cystic Fibrosis Foundation state that the use of the HbA1c as a screening tool is not recommended [9].

As advancements in CF therapies occur, survival rates are steadily improving, with many patients expected to live beyond the age of 50 years. However, as survival improves, additional complications, such as CFRD and its preceding dysglycemic states, become increasingly prevalent. Accurate and early detection of at-risk patients is critical for improving long-term outcomes [2]. Some studies have suggested that insulin treatment may be beneficial in dysglycemic states [21, 22]. Additional studies have reported that early detection of dysglycemic states, even without insulin treatment, may slow the decline of pulmonary function and nutritional status through education and nutrition counseling [13]. Further studies are required to determine more efficient and effective methods than the 2-hour OGTT to identify these pre-CFRD states. The optimal treatment for dysglycemia has not been established and future research is required to address this issue.

There are some limitations in this study. One limitation was its retrospective design. As well, despite extensively reviewing electronic medical records during a 22-year period, we identified only a relatively low number of patients with complete OGTT results (glucose measured at all time points) within the 90-day timeframe of an HbA1c measurement. For many patients, only partial OGTT results were available. Additionally, the number of patients was low in each of the IGT, INDET, or IFG groups. Another limitation was that the chart review encompassed a long time period (1997 to 2019), during which a wide variety of CF treatment advancements may have impacted the results. Finally, the addition of a pediatric endocrinologist to the UMass pediatric CF team in March 2017 skewed the results towards the pediatric population who may or may not have yet developed significant dysglycemia or CFRD.

Despite these limitations, our findings suggest that HbA1c is an ineffective screening tool for identifying CF patients at risk for IGT, INDET, or IGT. Thus, further studies are warranted to evaluate other approaches to more efficiently diagnose pre-CFRD dysglycemia. Less complicated testing than the 2-hour OGTT will reduce the annual burden on these patients, potentially leading to earlier diagnosis and treatment.

## Supporting information

S1 Data (DOCX)
