# Supplementary material for: Use of hemoglobin A1c to identify dysglycemia in cystic fibrosis
Source: PLoS One. 2021 Apr 21;16(4):e0250036. doi: 10.1371/journal.pone.0250036 (PMC8059836; doi:10.1371/journal.pone.0250036)
Supplement: S1 Data — (DOCX) [file pone.0250036.s001.docx]

S1 Data.

Table 1: Subject Data

| **PATIENT_GENDER** | **PATIENT_RACE** | **PATIENT_ETHNICITY** | **PANCREATIC_INS** | **DATE_DIFF** | **AGE_AT_HA1C** | **HA1C** | **GTT_0HR** | **GTT_1HR** | **GTT_2HR** | **CRP** | **ESR** |
| --- | --- | --- | --- | --- | --- | --- | --- | --- | --- | --- | --- |
| Male | White | Not Hispanic or Latino | 1 | -26 | 31.00 | 5.30 | 97.00 | 226.00 | 201.00 |  |  |
| Male | White | Not Hispanic or Latino | 1 | 0 | 17.00 | 6.00 | 93.00 |  | 95.00 |  |  |
| Male | White | Not Hispanic or Latino | 1 | 67 | 20.00 | 5.40 | 104.00 | 184.00 | 152.00 |  |  |
| Female | White | Not Hispanic or Latino | 1 | -50 | 15.00 | 5.70 | 200.00 |  |  | <1.0 |  |
| Female | White | Not Hispanic or Latino | 1 | 7 | 16.00 | 5.80 | 100.00 |  | 130.00 | <1.0 |  |
| Female | White | Not Hispanic or Latino | 1 | 54 | 18.00 | 5.40 |  | 105.00 | 168.00 | <1.0 |  |
| Female | White | Not Hispanic or Latino | 1 | 1 | 18.00 | 4.90 |  | 105.00 | 168.00 | <1.0 |  |
| Male | White | Not Hispanic or Latino | 1 | -77 | 18.00 | 5.00 | 95.00 | 123.00 | 92.00 | <1.0 | 9 |
| Male | White | Not Hispanic or Latino | 0 | 0 | 13.00 | 4.80 | 107.00 | 88.00 | 120.00 |  |  |
| Male | White | NULL | 1 | 13 | 17.00 | 6.00 | 99.00 |  |  | 33.6 | 43 |
| Male | White | NULL | 1 | 10 | 17.00 | 6.10 | 99.00 |  |  | 33.6 | 43 |
| Male | White | NULL | 1 | 8 | 19.00 | 5.70 | 91.00 |  | 211.00 | 33.6 | 43 |
| Male | White | NULL | 1 | 4 | 19.00 | 5.80 | 91.00 |  | 211.00 | 33.6 | 43 |
| Female | White | Not Hispanic or Latino | 1 | 0 | 16.00 | 5.20 | 104.00 | 397.00 | 218.00 | 1.1 | 12 |
| Female | White | Not Hispanic or Latino | 0 | 10 | 17.00 | 5.20 | 86.00 | 178.00 | 67.00 |  |  |
| Male | White | Not Hispanic or Latino | 1 | 0 | 36.00 | 5.40 | 112.00 | 226.00 | 94.00 |  |  |
| Male | White | Not Hispanic or Latino | 0 | 11 | 15.00 | 5.50 | 86.00 | 132.00 | 115.00 | <1.0 |  |
| Male | White | NULL | 0 | 10 | 18.00 | 5.80 | 86.00 |  | 112.00 |  | 17 |
| Male | White | NULL | 1 | 2 | 21.00 | 7.00 | 258.00 |  | 260.00 |  |  |
| Female | Other | Hispanic or Latino | 1 | 11 | 17.00 | 4.80 | 84.00 |  | 103.00 | 217.9 | 105 |
| Female | Other | Hispanic or Latino | 1 | 7 | 17.00 | 5.00 | 89.00 |  | 103.00 | 217.9 | 105 |
| Female | Other | Hispanic or Latino | 1 | 85 | 18.00 | 4.70 | 95.00 | 210.00 | 64.00 | 217.9 | 105 |
| Female | Other | Hispanic or Latino | 1 | 12 | 18.00 | 4.70 | 95.00 | 210.00 | 64.00 | 217.9 | 105 |
| Female | Other | Hispanic or Latino | 1 | 13 | 19.00 | 5.10 | 120.00 | 265.00 | 169.00 | 217.9 | 105 |
| Female | White | Not Hispanic or Latino | 1 | 7 | 16.00 | 5.30 | 78.00 |  | 112.00 | 2.1 |  |
| Female | White | Not Hispanic or Latino | 1 | 9 | 17.00 | 5.10 | 89.00 | 180.00 | 116.00 | 2.1 |  |
| Female | White | Not Hispanic or Latino | 1 | 13 | 22.00 | 5.30 | 174.00 | 99.00 | 134.00 | 2.8 |  |
| Female | White | Not Hispanic or Latino | 1 | 11 | 23.00 | 5.30 | 84.00 | 168.00 | 64.00 | 2.8 |  |
| Female | White | Not Hispanic or Latino | 1 | -12 | 21.00 | 6.50 | 79.00 | 220.00 | 217.00 | 139.9 | 61 |
| Female | White | Not Hispanic or Latino | 1 | 0 | 29.00 | 6.00 | 78.00 | 206.00 | 158.00 | 396.7 | 57 |
| Male | White | Not Hispanic or Latino | 1 | 12 | 38.00 | 5.50 | 119.00 | 243.00 | 162.00 | 1.1 |  |
| Female | White | NULL | 0 | 29 | 15.00 | 5.70 | 94.00 | 180.00 | 151.00 | 11.2 | 57 |
| Female | Hispanic | Hispanic or Latino | 0 | 34 | 19.00 | 5.10 | 89.00 | 133.00 | 85.00 |  |  |
| Male | White | Not Hispanic or Latino | 1 | 2 | 16.00 | 5.80 | 75.00 | 153.00 | 100.00 |  |  |
| Male | White | Not Hispanic or Latino | 1 | -1 | 17.00 | 5.60 | 88.00 |  | 90.00 |  |  |
| Male | White | Not Hispanic or Latino | 1 | 60 | 18.00 | 5.80 | 100.00 | 263.00 | 61.00 |  |  |
| Male | White | Not Hispanic or Latino | 1 | 0 | 18.00 | 4.70 | 97.00 | 78.00 | 63.00 |  | 4 |
| Male | White | Not Hispanic or Latino | 1 | 75 | 7.00 | 5.00 | 84.00 | 88.00 | 85.00 | 1.9 |  |
| Female | White | Not Hispanic or Latino | 1 | 75 | 14.00 | 4.60 | 83.00 | 106.00 | 85.00 | <1.0 |  |
| Female | White | Not Hispanic or Latino | 0 | 75 | 15.00 | 5.20 | 86.00 | 83.00 | 56.00 |  |  |
| Male | White | Not Hispanic or Latino | 0 | -35 | 63.00 | 6.70 | 94.00 |  | 196.00 | 2.8 |  |
| Male | White | Not Hispanic or Latino | 1 | 12 | 16.00 | 5.40 | 108.00 | 279.00 | 261.00 | <1.0 | 14 |
| Male | White | Not Hispanic or Latino | 1 | 76 | 16.00 | 5.40 | 99.00 | 175.00 | 96.00 | <1.0 | 14 |
| Male | White | Not Hispanic or Latino | 1 | 13 | 15.00 | 5.60 | 111.00 | 179.00 | 149.00 | 2.3 |  |
| Male | White | Not Hispanic or Latino | 1 | 9 | 17.00 | 6.00 | 142.00 | 153.00 | 123.00 | 2.3 |  |
| Male | White | Not Hispanic or Latino | 1 | -72 | 40.00 | 5.90 |  |  | 230.00 | 58.4 |  |
| Male | White | Not Hispanic or Latino | 1 | -3 | 41.00 | 6.00 |  |  | 276.00 | 58.4 |  |
| Female | White | Not Hispanic or Latino | 1 | 11 | 21.00 | 6.70 | 89.00 |  | 139.00 | 28.8 | 84 |
| Female | White | Not Hispanic or Latino | 1 | 11 | 21.00 | 5.60 | 79.00 | 167.00 | 201.00 | 28.8 | 84 |
| Female | White | Not Hispanic or Latino | 1 | -49 | 22.00 | 6.10 | 79.00 | 167.00 | 201.00 | 28.8 | 84 |
| Male | White | Not Hispanic or Latino | 1 | -6 | 16.00 | 5.60 | 97.00 |  | 74.00 | <1.0 |  |
| Male | White | Not Hispanic or Latino | 1 | -27 | 16.00 | 5.50 | 97.00 |  | 74.00 | <1.0 |  |
| Male | White | Not Hispanic or Latino | 1 | 39 | 18.00 | 5.10 | 107.00 | 160.00 | 50.00 | <1.0 |  |
| Male | White | Not Hispanic or Latino | 1 | -13 | 9.00 | 5.60 | 110.00 | 275.00 | 214.00 | <1.0 |  |
| Male | White | Not Hispanic or Latino | 1 | -34 | 9.00 | 5.40 | 110.00 | 275.00 | 214.00 | <1.0 |  |
| Male | White | Not Hispanic or Latino | 1 | -53 | 9.00 | 5.20 | 110.00 | 275.00 | 214.00 | <1.0 |  |
| Male | White | Not Hispanic or Latino | 1 | 85 | 16.00 | 5.60 | 99.00 | 227.00 | 55.00 | 5 |  |
| Male | White | Not Hispanic or Latino | 1 | 0 | 24.00 | 5.50 |  | 190.00 | 129.00 | <1.0 |  |
| Male | White | Not Hispanic or Latino | 1 | 0 | 25.00 | 5.50 |  | 141.00 | 69.00 | <1.0 |  |
| Male | White | Not Hispanic or Latino | 1 | -77 | 25.00 | 5.40 |  | 141.00 | 69.00 | <1.0 |  |
| Female | White | Not Hispanic or Latino | 1 | 13 | 29.00 | 6.00 |  |  | 175.00 | 1.8 |  |
| Female | Unknown, White | Not Hispanic or Latino | 1 | -1 | 26.00 | 6.50 | 87.00 |  | 230.00 | 3.2 |  |
| Male | White | Not Hispanic or Latino | 1 | 0 | 35.00 | 4.50 |  | 285.00 | 196.00 | 30.9 | 36 |
| Male | White | Not Hispanic or Latino | 1 | -39 | 35.00 | 4.70 |  | 285.00 | 196.00 | 30.9 | 36 |
| Male | White | Not Hispanic or Latino | 1 | 9 | 7.00 | 5.20 | 87.00 | 161.00 | 121.00 | <1.0 |  |
| Female | White | Not Hispanic or Latino | 0 | 9 | 10.00 | 5.50 | 89.00 | 163.00 | 133.00 | <1.0 |  |
| Male | White | Not Hispanic or Latino | 1 | 1 | 20.00 | 5.40 | 95.00 | 144.00 | 51.00 |  |  |
| Male | White | Not Hispanic or Latino | 1 | 0 | 21.00 | 5.90 | 105.00 | 249.00 | 103.00 |  |  |
| Female | White | Not Hispanic or Latino | 0 | 0 | 20.00 | 5.80 | 84.00 |  | 139.00 |  |  |
| Male | White | Not Hispanic or Latino | 1 | -63 | 7.00 | 5.60 | 101.00 | 152.00 | 107.00 | <1.0 |  |
| Male | Black or African American | NULL | 0 | 10 | 24.00 | 5.40 |  |  | 155.00 |  |  |
| Male | White | Not Hispanic or Latino | 1 | -67 | 22.00 | 5.70 | 97.00 | 197.00 | 113.00 | 5.4 | 32 |
| Male | White | Not Hispanic or Latino | 1 | 11 | 23.00 | 6.00 |  | 353.00 | 127.00 | 5.4 | 32 |
| Male | White | Not Hispanic or Latino | 1 | -39 | 23.00 | 5.80 |  | 353.00 | 127.00 | 5.4 | 32 |
| Male | White | Not Hispanic or Latino | 1 | 4 | 23.00 | 5.70 | 95.00 | 263.00 | 168.00 | 5.4 | 32 |
| Male | White | Not Hispanic or Latino | 0 | 0 | 41.00 | 6.80 | 171.00 |  |  |  |  |
| Female | White | Not Hispanic or Latino | 0 | 0 | 46.00 | 6.10 | 84.00 |  | 79.00 | 8.3 |  |
| Female | White | Not Hispanic or Latino | 0 | 0 | 47.00 | 5.80 | 94.00 |  | 162.00 | 8.3 |  |
| Female | White | Not Hispanic or Latino | 1 | 10 | 10.00 | 5.80 | 157.00 |  | 126.00 | <1.0 |  |
| Female | White | Not Hispanic or Latino | 1 | 11 | 11.00 | 5.90 | 99.00 | 213.00 | 80.00 | <1.0 |  |
| Female | White | Not Hispanic or Latino | 1 | 49 | 12.00 | 5.40 | 89.00 | 229.00 | 103.00 | <1.0 |  |
| Female | White | Not Hispanic or Latino | 1 | -67 | 12.00 | 5.60 | 89.00 | 229.00 | 103.00 | <1.0 |  |
| Male | White | NULL | 0 | 0 | 20.00 | 6.70 | 69.00 | 197.00 | 212.00 |  | 10 |
| Male | White | Not Hispanic or Latino | 1 | 2 | 21.00 | 5.70 | 82.00 |  | 103.00 | 1.3 |  |

Data 2: Statistics and Analysis

| **Row Labels** | **Count of PATIENT_GENDER** | 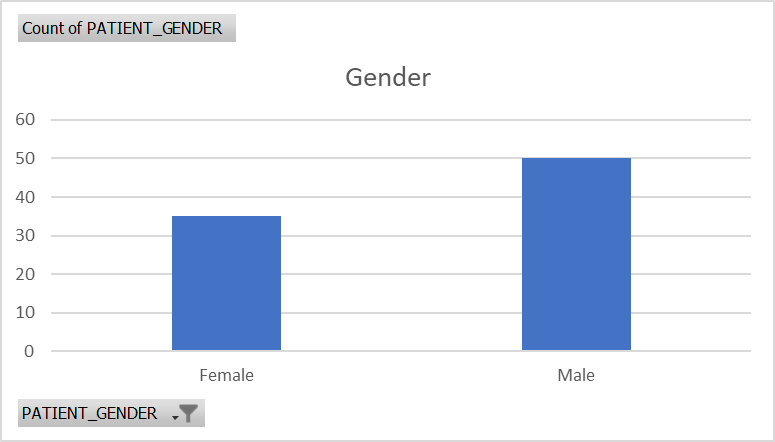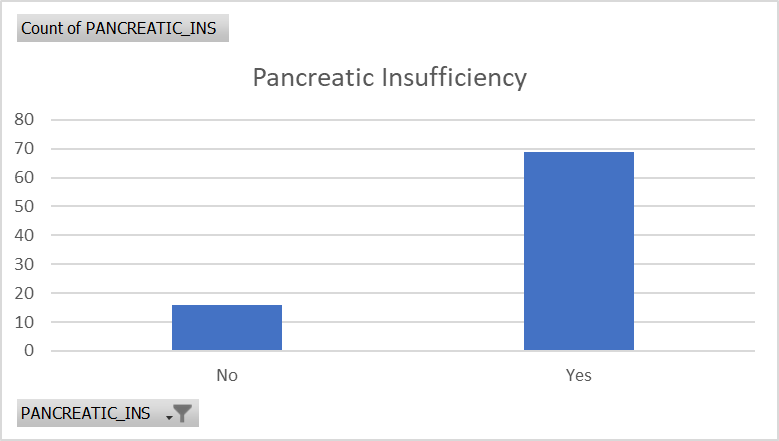   \|  \| \| --- \| |  |  |  | 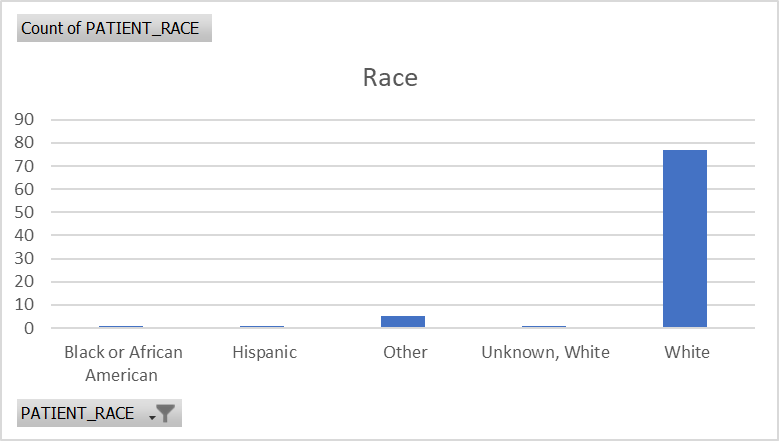 |  |  |  |  |  |  |  |  |  |  |  |  |  |  |  |  |  |  |
| --- | --- | --- | --- | --- | --- | --- | --- | --- | --- | --- | --- | --- | --- | --- | --- | --- | --- | --- | --- | --- | --- | --- | --- | --- | --- |
| Female | 35 |  |  |  |  |  |  |  |  |  |  |  |  |  |  |  |  |  |  |  |  |  |  |  |
| Male | 50 |  |  |  |  |  |  |  |  |  |  |  |  |  |  |  |  |  |  |  |  |  |  |  |
| **Grand Total** | **85** |  |  |  |  |  |  |  |  |  |  |  |  |  |  |  |  |  |  |  |  |  |  |  |
|  |  |  |  |  |  |  |  |  |  |  |  |  |  |  |  |  |  |  |  |  |  |  |  |  |
|  |  |  |  |  |  |  |  |  |  |  |  |  |  |  |  |  |  |  |  |  |  |  |  |  |
| **Row Labels** | **Count of PATIENT_RACE** |  |  |  |  |  |  |  |  |  |  |  |  |  |  |  |  |  |  |  |  |  |  |  |
| Black or African American | 1 |  |  |  |  | 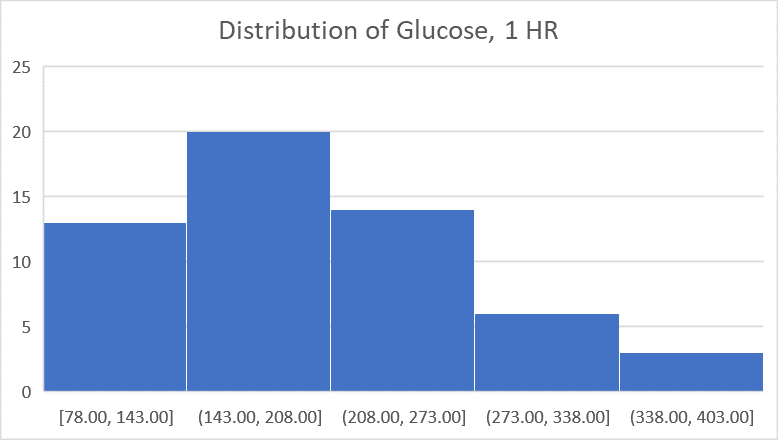 |  |  |  |  |  |  |  |  |  |  |  |  |  |  |  |  |  |  |
| Hispanic | 1 | 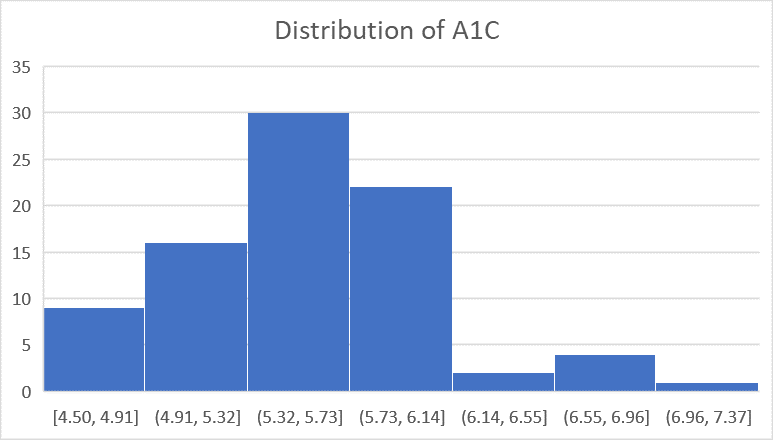 |  |  |  |  |  |  |  |  |  |  |  |  |  |  |  |  |  |  |  |  |  |  |
| Other | 5 |  |  |  |  |  |  |  |  |  |  |  |  |  |  |  |  |  |  |  |  |  |  |  |
| Unknown, White | 1 |  |  |  |  |  |  |  |  |  |  |  |  |  |  |  |  |  |  |  |  |  |  |  |
| White | 77 |  |  |  |  |  |  |  |  |  |  |  |  |  |  |  |  |  |  |  |  |  |  |  |
| **Grand Total** | **85** |  |  |  |  |  |  |  |  |  |  |  |  |  |  |  |  |  |  |  |  |  |  |  |
|  |  |  |  |  |  |  |  |  |  |  |  |  |  |  |  |  |  |  |  |  |  |  |  |  |
| **Row Labels** | **Count of PANCREATIC_INS** | 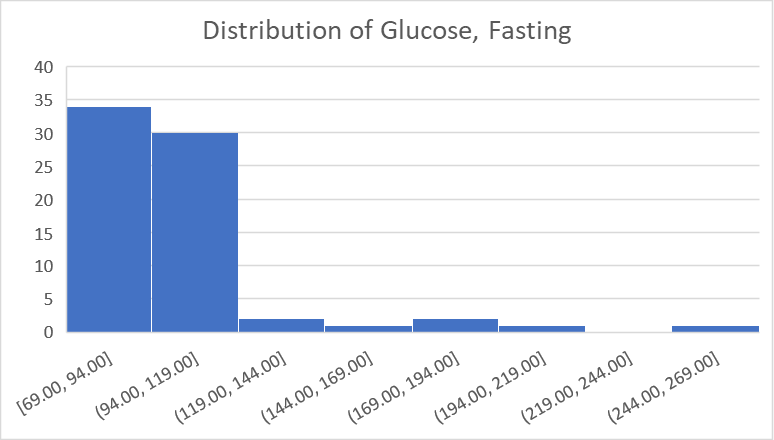 | | | | | | | | | | | | | | | | | 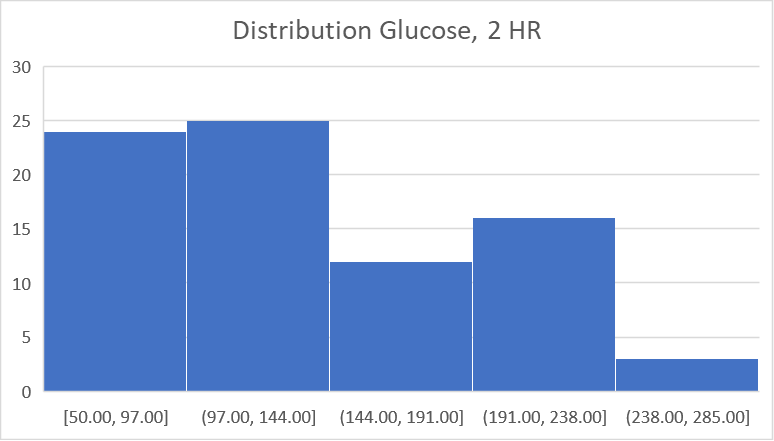 | | | | | |
| No | 16 |  |  |  |  |  |  |  |  |  |  |  |  |  |  |  |  |  |  |  |  |  |  |  |
| Yes | 69 |  |  |  |  |  |  |  |  |  |  |  |  |  |  |  |  |  |  |  |  |  |  |  |
| **Grand Total** | **85** |  |  |  |  |  |  |  |  |  |  |  |  |  |  |  |  |  |  |  |  |  |  |  |
|  |  |  |  |  |  |  |  |  |  |  |  |  |  |  |  |  |  |  |  |  |  |  |  |  |
|  |  |  |  |  |  |  |  |  |  |  |  |  |  |  |  |  |  |  |  |  |  |  |  |  |
|  |  |  |  |  |  |  |  |  |  |  |  |  |  |  |  |  |  |  |  |  |  |  |  |  |
|  | 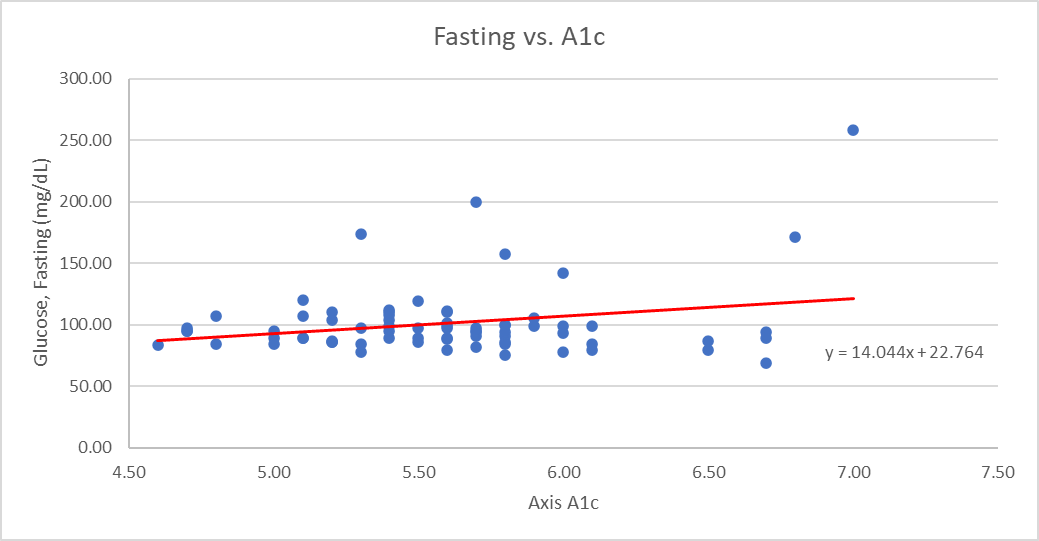 |  |  |  |  |  |  |  |  |  |  |  |  |  |  |  |  |  |  |  |  |  |  |  |
|  |  |  |  |  |  |  |  |  |  |  |  |  |  |  |  |  |  |  |  |  |  |  |  |  |
|  |  |  |  |  |  |  |  |  |  |  |  |  |  |  |  |  |  |  |  |  |  |  |  |  |
|  |  |  |  |  |  |  |  |  |  |  |  |  |  |  |  |  |  |  |  |  |  |  |  |  |
|  |  |  |  |  |  |  |  |  |  |  |  |  |  |  |  |  |  |  |  |  |  |  |  |  |
|  |  |  |  |  |  |  |  |  |  |  |  |  |  |  |  |  |  |  |  |  |  |  |  |  |
|  |  |  |  |  |  |  |  |  |  |  |  |  |  |  |  |  |  |  |  |  |  |  |  |  |
|  |  |  |  |  |  |  |  |  |  |  |  |  |  |  |  |  |  |  |  |  |  |  |  |  |
|  |  |  |  |  |  |  |  |  |  |  |  |  |  |  |  |  |  |  |  |  |  |  |  |  |
| **PEARSON CORRELATION** | |  |  |  |  |  |  |  |  |  |  |  |  |  |  |  |  |  |  |  |  |  |  |  |
|  | *AGE_AT_HA1C* | *HA1C* | *GTT_0HR* | *GTT_1HR* | *GTT_2HR* |  |  |  |  |  |  |  |  |  |  |  |  |  |  |  |  |  |  |  |
| AGE_AT_HA1C | 1 |  |  |  |  |  |  |  |  |  |  |  |  |  |  |  |  |  |  |  |  |  |  |  |
| HA1C | 0.312305916 | 1 |  |  |  |  |  | | | | | | |  |  |  |  |  |  |  |  |  |  |  |
| GTT_0HR | 0.0418452 | 0.24841926 | 1 |  |  |  |  | | | | | | | | | | | |  |  |  |  |  |  |
| GTT_1HR | 0.228075134 | 0.21932477 | 0.10372159 | 1 |  |  |  |  |  |  |  |  |  |  |  |  |  |  |  |  |  |  |  |  |
| GTT_2HR | 0.2763835 | 0.36913035 | 0.2546624 | 0.45345581 | 1 |  |  |  |  |  |  |  |  |  |  |  |  |  |  |  |  |  |  |  |
|  |  |  |  |  |  |  |  |  |  |  |  |  |  |  |  |  |  |  |  |  |  |  |  |  |
|  |  |  |  |  |  |  |  |  |  |  |  |  |  |  |  |  |  |  |  |  |  |  |  |  |
| **FASTING VS A1C Regression** | |  |  |  |  |  |  |  |  |  |  |  |  |  |  |  |  |  |  |  |  |  |  |  |
| SUMMARY OUTPUT | |  |  |  |  |  |  |  |  |  |  |  |  |  |  |  |  |  |  |  |  |  |  |  |
|  |  |  |  |  |  |  |  |  |  | | | | | | | |  |  |  |  |  |  |  |  |
| *Regression Statistics* | |  |  |  |  |  |  |  |  |  |  |  |  |  |  |  |  |  |  |  |  |  |  |  |
| Multiple R | 0.248419263 |  | Fasting p = 0.037, which is less than .05. There is a relationship | | | | |  |  |  |  |  |  |  |  |  |  |  |  |  |  |  |  |  |
| R Square | 0.06171213 |  | with A1c. However, the R squared is VERY small, meaning that | | | | |  |  |  |  |  |  |  |  |  |  |  |  |  |  |  |  |  |
| Adjusted R Square | 0.048113755 |  | it is not a strong relationship. | | |  |  |  |  |  |  |  |  |  |  |  |  |  |  |  |  |  |  |  |
| Standard Error | 0.506511059 |  |  |  |  |  |  |  |  |  |  |  |  |  |  |  |  |  |  |  |  |  |  |  |
| Observations | 71 |  |  |  |  |  |  |  |  |  |  |  |  |  |  |  |  |  |  |  |  |  |  |  |
|  |  |  |  |  |  |  |  |  |  |  |  |  |  |  |  |  |  |  |  |  |  |  |  |  |
| ANOVA |  |  |  |  |  |  |  |  |  |  |  |  |  |  |  |  |  |  |  |  |  |  |  |  |
|  | *df* | *SS* | *MS* | *F* | *Significance F* |  |  |  |  |  |  |  |  |  |  |  |  |  |  |  |  |  |  |  |
| Regression | 1 | 1.1642906 | 1.1642906 | 4.53819889 | 0.03671397 |  |  |  |  |  |  |  |  |  |  |  |  |  |  |  |  |  |  |  |
| Residual | 69 | 17.7021883 | 0.25655345 |  |  |  |  |  |  |  |  |  |  |  |  |  |  |  |  |  |  |  |  |  |
| Total | 70 | 18.8664789 |  |  |  |  |  |  |  |  |  |  |  |  |  |  |  |  |  |  |  |  |  |  |
|  |  |  |  |  |  |  |  |  |  |  |  |  |  |  |  |  |  |  |  |  |  |  |  |  |
|  | *Coefficients* | *Standard Error* | *t Stat* | *P-value* | *Lower 95%* | *Upper 95%* | *Lower 95.0%* | *Upper 95.0%* |  |  |  |  |  |  |  |  |  |  |  |  |  |  |  |  |
| Intercept | 5.147772158 | 0.21745111 | 23.6732393 | 7.6242E-35 | 4.71396906 | 5.58157525 | 4.71396906 | 5.58157525 |  |  |  |  |  |  |  |  |  |  |  |  |  |  |  |  |
| GTT_0HR | 0.004394297 | 0.00206275 | 2.13030488 | 0.03671397 | 0.00027921 | 0.00850938 | 0.00027921 | 0.00850938 |  |  |  |  |  |  |  |  |  |  |  |  |  |  |  |  |
|  |  |  |  |  |  |  |  |  |  |  |  |  |  |  |  |  |  |  |  |  |  |  |  |  |
|  |  |  |  |  |  |  |  |  |  |  |  |  |  |  |  |  |  |  |  |  |  |  |  |  |
| **OGTT 1 HR vs A1c Regression** | |  |  |  |  |  |  |  |  |  |  |  |  |  |  |  |  |  |  |  |  |  |  |  |
| SUMMARY OUTPUT | |  |  |  |  |  |  |  |  | | | | | | | |  |  |  |  |  |  |  |  |
|  |  |  |  |  |  |  |  |  |  |  |  |  |  |  |  |  |  |  |  |  |  |  |  |  |
| *Regression Statistics* | |  |  |  |  |  |  |  |  |  |  |  |  |  |  |  |  |  |  |  |  |  |  |  |
| Multiple R | 0.219324766 |  | Fasting p = 0.104, which is not less than .05. There is not a relationship | | | | | |  |  |  |  |  |  |  |  |  |  |  |  |  |  |  |  |
| R Square | 0.048103353 |  | with A1c. The R squared is VERY small, meaning that | | | |  |  |  |  |  |  |  |  |  |  |  |  |  |  |  |  |  |  |
| Adjusted R Square | 0.030475637 |  | 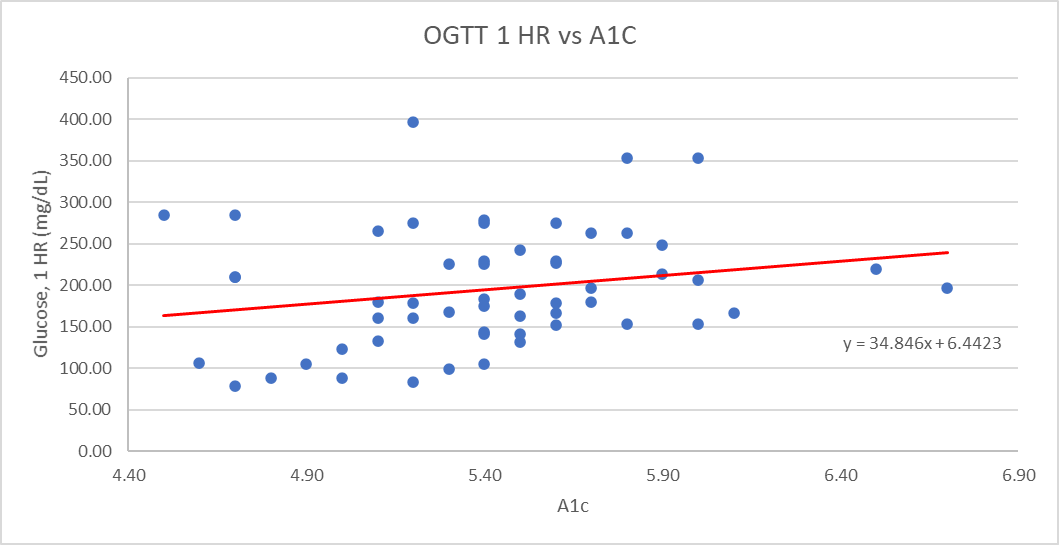most of the data cannot be explained by a linear relationship. | | | | |  |  |  |  |  |  |  |  |  |  |  |  |  |  |  |  |  |
| Standard Error | 0.438914285 |  |  |  |  |  |  |  |  |  |  |  |  |  |  |  |  |  |  |  |  |  |  |  |
| Observations | 56 |  |  |  |  |  |  |  |  |  |  |  |  |  |  |  |  |  |  |  |  |  |  |  |
|  |  |  |  |  |  |  |  |  |  |  |  |  |  |  |  |  |  |  |  |  |  |  |  |  |
| ANOVA |  |  |  |  |  |  |  |  |  |  |  |  |  |  |  |  |  |  |  |  |  |  |  |  |
|  | *df* | *SS* | *MS* | *F* | *Significance F* |  |  |  |  |  |  |  |  |  |  |  |  |  |  |  |  |  |  |  |
| Regression | 1 | 0.52570093 | 0.52570093 | 2.72884779 | 0.10435433 |  |  |  |  |  |  |  |  |  |  |  |  |  |  |  |  |  |  |  |
| Residual | 54 | 10.4028705 | 0.19264575 |  |  |  |  |  |  |  |  |  |  |  |  |  |  |  |  |  |  |  |  |  |
| Total | 55 | 10.9285714 |  |  |  |  |  |  |  |  |  |  |  |  |  |  |  |  |  |  |  |  |  |  |
|  |  |  |  |  |  |  |  |  |  |  |  |  |  |  |  |  |  |  |  |  |  |  |  |  |
|  | *Coefficients* | *Standard Error* | *t Stat* | *P-value* | *Lower 95%* | *Upper 95%* | *Lower 95.0%* | *Upper 95.0%* |  |  |  |  |  |  |  |  |  |  |  |  |  |  |  |  |
| Intercept | 5.144947063 | 0.17327424 | 29.6925093 | 3.9884E-35 | 4.79755312 | 5.492341 | 4.79755312 | 5.492341 |  |  |  |  |  |  |  |  |  |  |  |  |  |  |  |  |
| GTT_1HR | 0.001380465 | 0.00083567 | 1.65192245 | 0.10435433 | -0.000295 | 0.00305589 | -0.000295 | 0.00305589 |  |  |  |  |  |  |  |  |  |  |  |  |  |  |  |  |
|  |  |  |  |  |  |  |  |  |  |  |  |  |  |  |  |  |  |  |  |  |  |  |  |  |
|  |  |  |  |  |  |  |  |  |  |  |  |  |  |  |  |  |  |  |  |  |  |  |  |  |
| **OGTT 2 HR vs A1c Regression** | |  |  |  |  |  |  |  |  |  |  |  |  |  |  |  |  |  |  |  |  |  |  |  |
| SUMMARY OUTPUT | |  |  |  |  |  |  |  |  | | | | | | | |  |  |  |  |  |  |  |  |
|  |  |  |  |  |  |  |  |  |  |  |  |  |  |  |  |  |  |  |  |  |  |  |  |  |
| *Regression Statistics* | |  |  |  |  |  |  |  |  |  |  |  |  |  |  |  |  |  |  |  |  |  |  |  |
| Multiple R | 0.369130348 |  | Fasting p = 0.0008, which is less than .05. There is a relationship | | | | |  |  |  |  |  |  |  |  |  |  |  |  |  |  |  |  |  |
| R Square | 0.136257214 |  | with A1c. However, the R squared is VERY small, meaning that | | | | |  |  |  |  |  |  |  |  |  |  |  |  |  |  |  |  |  |
| Adjusted R Square | 0.125183588 |  | it is not a strong relationship. | | |  |  |  |  |  |  |  |  |  |  |  |  |  |  |  |  |  |  |  |
| Standard Error | 0.470807155 |  |  |  |  | 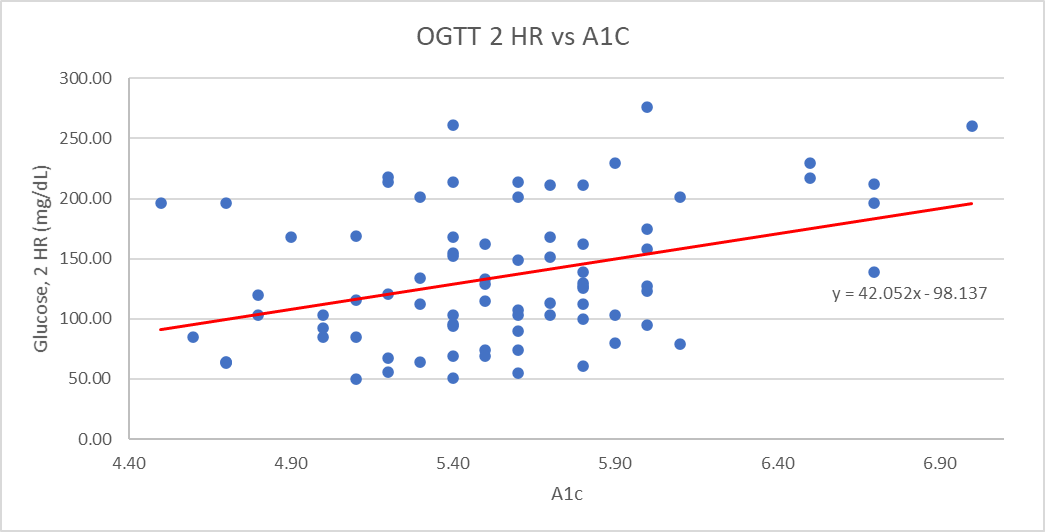 |  |  |  |  |  |  |  |  |  |  |  |  |  |  |  |  |  |  |
| Observations | 80 |  |  |  |  |  |  |  |  |  |  |  |  |  |  |  |  |  |  |  |  |  |  |  |
|  |  |  |  |  |  |  |  |  |  |  |  |  |  |  |  |  |  |  |  |  |  |  |  |  |
| ANOVA |  |  |  |  |  |  |  |  |  |  |  |  |  |  |  |  |  |  |  |  |  |  |  |  |
|  | *df* | *SS* | *MS* | *F* | *Significance F* |  |  |  |  |  |  |  |  |  |  |  |  |  |  |  |  |  |  |  |
| Regression | 1 | 2.72744361 | 2.72744361 | 12.3046616 | 0.00075312 |  |  |  |  |  |  |  |  |  |  |  |  |  |  |  |  |  |  |  |
| Residual | 78 | 17.2894314 | 0.22165938 |  |  |  |  |  |  |  |  |  |  |  |  |  |  |  |  |  |  |  |  |  |
| Total | 79 | 20.016875 |  |  |  |  |  |  |  |  |  |  |  |  |  |  |  |  |  |  |  |  |  |  |
|  |  |  |  |  |  |  |  |  |  |  |  |  |  |  |  |  |  |  |  |  |  |  |  |  |
|  | *Coefficients* | *Standard Error* | *t Stat* | *P-value* | *Lower 95%* | *Upper 95%* | *Lower 95.0%* | *Upper 95.0%* |  |  |  |  |  |  |  |  |  |  |  |  |  |  |  |  |
| Intercept | 5.106359411 | 0.13534604 | 37.7281768 | 7.4807E-52 | 4.83690614 | 5.37581268 | 4.83690614 | 5.37581268 |  |  |  |  |  |  |  |  |  |  |  |  |  |  |  |  |
| GTT_2HR | 0.00324023 | 0.00092372 | 3.5078001 | 0.00075312 | 0.00140124 | 0.00507922 | 0.00140124 | 0.00507922 |  |  |  |  |  |  |  |  |  |  |  |  |  |  |  |  |
|  |  |  |  |  |  |  |  |  |  |  |  |  |  |  |  |  |  |  |  |  |  |  |  |  |
|  |  |  |  |  |  |  |  |  |  |  |  |  |  |  |  |  |  |  |  |  |  |  |  |  |
| **MULTIPLE REGRESSION** | |  |  |  |  |  |  |  |  |  |  |  |  |  |  |  |  |  |  |  |  |  |  |  |
| **Since fasting and 2 HR were individual significant, let's look at them in combination** | | | | |  |  |  |  |  |  |  |  |  |  |  |  |  |  |  |  |  |  |  |  |
| SUMMARY OUTPUT | |  |  |  |  |  |  |  |  |  |  |  |  |  |  |  |  |  |  |  |  |  |  |  |
|  |  |  |  |  |  |  |  |  |  |  |  |  |  |  |  |  |  |  |  |  |  |  |  |  |
| *Regression Statistics* | |  |  |  |  |  |  |  |  |  |  |  |  |  |  |  |  |  |  |  |  |  |  |  |
| Multiple R | 0.468097504 |  | When looking at them together, only 2 HR has a significant effect on | | | | | |  |  |  |  |  |  |  |  |  |  |  |  |  |  |  |  |
| R Square | 0.219115273 |  | A1C with p =0.0003. This is intriguing as it suggests the 2 HR is the best way to look at how OGTT relates to a1c | | | | | | | | |  |  |  |  |  |  |  |  |  |  |  |  |  |
| Adjusted R Square | 0.194712626 |  | Again, the R squared is low, which means most of the data is not explained very well, so it's a weak relationship | | | | | | | | |  |  |  |  |  |  |  |  |  |  |  |  |  |
| Standard Error | 0.454111156 |  |  |  |  |  |  |  |  |  |  |  |  |  |  |  |  |  |  |  |  |  |  |  |
| Observations | 67 |  |  |  |  |  |  |  |  |  |  |  |  |  |  |  |  |  |  |  |  |  |  |  |
|  |  |  |  |  |  |  |  |  |  |  |  |  |  |  |  |  |  |  |  |  |  |  |  |  |
| ANOVA |  |  |  |  |  |  |  |  |  |  |  |  |  |  |  |  |  |  |  |  |  |  |  |  |
|  | *df* | *SS* | *MS* | *F* | *Significance F* |  |  |  |  |  |  |  |  |  |  |  |  |  |  |  |  |  |  |  |
| Regression | 2 | 3.70330975 | 1.85165488 | 8.9791598 | 0.00036541 |  |  |  |  |  |  |  |  |  |  |  |  |  |  |  |  |  |  |  |
| Residual | 64 | 13.1978843 | 0.20621694 |  |  |  |  |  |  |  |  |  |  |  |  |  |  |  |  |  |  |  |  |  |
| Total | 66 | 16.901194 |  |  |  |  |  |  |  |  |  |  |  |  |  |  |  |  |  |  |  |  |  |  |
|  |  |  |  |  |  |  |  |  |  |  |  |  |  |  |  |  |  |  |  |  |  |  |  |  |
|  | *Coefficients* | *Standard Error* | *t Stat* | *P-value* | *Lower 95%* | *Upper 95%* | *Lower 95.0%* | *Upper 95.0%* |  |  |  |  |  |  |  |  |  |  |  |  |  |  |  |  |
| Intercept | 4.892967517 | 0.23170739 | 21.1170108 | 1.5234E-30 | 4.43007879 | 5.35585625 | 4.43007879 | 5.35585625 |  |  |  |  |  |  |  |  |  |  |  |  |  |  |  |  |
| GTT_0HR | 0.001511592 | 0.00220664 | 0.68501952 | 0.49580517 | -0.0028967 | 0.00591986 | -0.0028967 | 0.00591986 |  |  |  |  |  |  |  |  |  |  |  |  |  |  |  |  |
| GTT_2HR | 0.003977323 | 0.00102782 | 3.86966435 | 0.00025808 | 0.00192401 | 0.00603063 | 0.00192401 | 0.00603063 |  |  |  |  |  |  |  |  |  |  |  |  |  |  |  |  |

| **HA1C** | | **GTT_0HR** | | **GTT_1HR** | | **GTT_2HR** | |  | |  | |  | |  |  |  |  |  |
| --- | --- | --- | --- | --- | --- | --- | --- | --- | --- | --- | --- | --- | --- | --- | --- | --- | --- | --- |
| 4.70 | | 95.00 | | 210.00 | | 64.00 | |  | |  | |  | |  |  |  |  |  |
| 4.70 | | 95.00 | | 210.00 | | 64.00 | |  | |  | |  | |  |  |  |  |  |
| 5.80 | | 100.00 | | 263.00 | | 61.00 | |  | |  | |  | |  |  |  |  |  |
| 5.60 | | 99.00 | | 227.00 | | 55.00 | |  | |  | |  | |  |  |  |  |  |
| 5.90 | | 99.00 | | 213.00 | | 80.00 | |  | |  | |  | |  |  |  |  |  |
| 5.40 | | 89.00 | | 229.00 | | 103.00 | |  | |  | |  | |  |  |  |  |  |
| 5.60 | | 89.00 | | 229.00 | | 103.00 | |  | |  | |  | |  |  |  |  |  |
|  | |  | |  | |  | |  | |  | |  | |  |  |  |  |  |
| *** INDET: Fasting < 100, 1 HR > 200, 2 HR < 140 | | | | | | | |  | |  | |  | |  |  |  |  |  |
|  | |  | |  | |  | |  | |  | |  | |  |  |  |  |  |
| only n = 7 | |  | |  | |  | |  | |  | |  | |  |  |  |  |  |
| Very small sample size!!! | | | |  | |  | |  | |  | |  | |  |  |  |  |  |
|  | |  | |  | |  | |  | |  | |  | |  |  |  |  |  |
|  | |  | |  | |  | |  | |  | |  | |  |  |  |  |  |
| SUMMARY OUTPUT | | | |  | |  | |  | |  | |  | |  |  |  |  |  |
|  | |  | |  | |  | |  | |  | |  | |  |  |  |  |  |
| *Regression Statistics* | | | |  | |  | |  | |  | |  | |  |  |  |  |  |
| Multiple R | | 0.93053332 | |  | |  | |  | |  | | | | | | | | |
| R Square | | 0.86589226 | |  | |  | |  | |  | | | | |  |  |  |  |
| Adjusted R Square | | 0.73178451 | |  | |  | |  | |  | | | | | | | | |
| Standard Error | | 0.25622048 | |  | |  | |  | |  | |  | |  |  |  |  |  |
| Observations | | 7 | |  | |  | |  | |  | |  | |  |  |  |  |  |
|  | |  | |  | |  | |  | |  | |  | |  |  |  |  |  |
| ANOVA | |  | |  | |  | |  | |  | |  | |  |  |  |  |  |
|  | | *df* | | *SS* | | *MS* | | *F* | | *Significance F* | |  | |  |  |  |  |  |
| Regression | | 3 | | 1.27162463 | | 0.42387488 | | 6.4566909 | | 0.07993466 | |  | |  |  |  |  |  |
| Residual | | 3 | | 0.1969468 | | 0.06564893 | |  | |  | |  | |  |  |  |  |  |
| Total | | 6 | | 1.46857143 | |  | |  | |  | |  | |  |  |  |  |  |
|  | |  | |  | |  | |  | |  | |  | |  |  |  |  |  |
|  | | *Coefficients* | | *Standard Error* | | *t Stat* | | *P-value* | | *Lower 95%* | | *Upper 95%* | | *Lower 95.0%* | *Upper 95.0%* |  |  |  |
| Intercept | | -12.611651 | | 4.51239798 | | -2.7948889 | | 0.06814143 | | -26.972115 | | 1.74881332 | | -26.972115 | 1.74881332 |  |  |  |
| GTT_0HR | | 0.13990808 | | 0.04294272 | | 3.25801623 | | 0.04720232 | | 0.00324518 | | 0.27657099 | | 0.00324518 | 0.27657099 |  |  |  |
| GTT_1HR | | 0.00969091 | | 0.00590929 | | 1.6399439 | | 0.19954987 | | -0.0091151 | | 0.02849692 | | -0.0091151 | 0.02849692 |  |  |  |
| GTT_2HR | | 0.03298386 | | 0.00970883 | | 3.39730595 | | 0.04254551 | | 0.00208604 | | 0.06388168 | | 0.00208604 | 0.06388168 |  |  |  |
|  | |  | |  | |  | |  | |  | |  | |  |  |  |  |  |
|  | |  | |  | |  | |  | |  | |  | |  |  |  |  |  |
| **Fasting and 2 HR have a significant effect on A1c (p = 0.047 and p = 0.042, respectively)** | | | | | | | | | | | | |  |  |  |  |  |  |
| **However, 1 HR does not (p = 0.200)** | | | | |  | |  | |  | |  |  |  |  |  |  |  |  |
| **R square is at about 86%, which means 86% of A1C can be explained by the OGTT** | | | | | | | | | | | | |  |  |  |  |  |  |
|  |  | |  | |  | |  | |  | |  |  |  |  |  |  |  |  |

Information Data 3: Methods and Results

| **Methods** |  |  |  |  |  |  |  |  |  |  |
| --- | --- | --- | --- | --- | --- | --- | --- | --- | --- | --- |
|  |  |  |  |  |  |  |  |  |  |  |
| Pearson correlations to identify how related the glucose levels are to the A1c |  |  |  |  |  |  |  |  |  |  |
| Univariate Linear Regressions for each stage of OGTT to identify individual effects on A1c |  |  |  |  |  |  |  |  |  |  |
| Multiple Linear regression to see how they perform together |  |  |  |  |  |  |  |  |  |  |
| All significance measure at p < 0.05 |  |  |  |  |  |  |  |  |  |  |
| Indeterminate glycemia identified within cohort |  |  |  |  |  |  |  |  |  |  |
| Multiple linear regression to see how OGTT relates to AIc |  |  |  |  |  |  |  |  |  |  |
|  |  |  |  |  |  |  |  |  |  |  |
| **Results** |  |  |  |  |  |  |  |  |  |  |
| PLEASE REFER TO TAB STATS AND RESULTS FOR PLOTS AND DESCRIPTIONS |  |  |  |  |  |  |  |  |  |  |
| n = 84 unique times an A1c and OGTT falls within our time frames |  |  |  |  |  |  |  |  |  |  |
| Some stages of OGTT missing |  |  |  |  |  |  |  |  |  |  |
| Individual linear regression show that fasting and 2 HR have a significant effect on A1c, but the relationship is weak | |  |  |  |  |  |  |  |  |  |
| Multiple regression using both of those shows 2 HR is the best predictor of A1c |  |  |  |  |  |  |  |  |  |  |
|  |  |  |  |  |  |  |  |  |  |  |
| Indeterminate glycemia: n = 7 (VERY SMALL sample size) |  |  |  |  |  |  |  |  |  |  |
| Multiple linear regression shows there is a relationship between Fasting and 2 HR glucose values in determining A1c  (see highlighted p values in other tab). There is not a significant relationship with 1 HR when in combination of the others) | | | | | | | | | | |
|  |  |  |  |  |  |  |  |  |  |  |
| **Discussion** |  |  |  |  |  |  |  |  |  |  |
| It looks like fasting and 2 HR values have provide a better understanding for the relationship with A1c than 1 hour.  The results are intriguing but need more sample size to know more. | | | | | | |  |  |  |  |
|  |  |  |  |  |  |  |  |  |  |  |
|  |  |  |  |  |  |  |  |  |  |  |
|  |  |  |  |  |  |  |  |  |  |  |
|  |  |  |  |  |  |  |  |  |  |  |
|  |  |  |  |  |  |  |  |  |  |  |
